# Caudate gray matter volumes and risk of relapse in Type A alcohol-dependent patients: A 7-year MRI follow-up study

**DOI:** 10.3389/fpsyt.2023.1067326

**Published:** 2023-02-15

**Authors:** Catherine Martelli, Eric Artiges, Rubén Miranda, Bruno Romeo, Amélie Petillion, Henri-Jean Aubin, Ammar Amirouche, Sandra Chanraud, Amine Benyamina, Jean-Luc Martinot

**Affiliations:** ^1^Institut National de la Santé et de la Recherche Médicale (INSERM) Research Unit 1299 “Trajectoires développementales en psychiatrie”, École Normale Supérieure Paris-Saclay, Université Paris-Saclay, Centre National de la Recherche Scientifique (CNRS) 9010, Centre Borelli, Gif-sur-Yvette, France; ^2^Department of Psychiatry and Addictology, Assistance Publique – Hôpitaux de Paris, Paul-Brousse Hospital, Villejuif, France; ^3^Psychiatry-Comorbidities-Addictions Research Unit (PSYCOMADD), Paris-Saclay University, Gif-sur-Yvette, France; ^4^Department of Psychiatry, Établissement Public de Santé (EPS) Barthélemy Durand, Etampes, France; ^5^Institut National de la Santé et de la Recherche Médicale Research Unit 1018, Centre de Recherche en Epidémiologie et Santé des Populations (CESP), Paris, France; ^6^Paris Sciences & Lettres (PSL) Research University-École Pratique des Hautes Études (EPHE), Paris, France; ^7^Institut de Neurosciences Cognitives et Intégratives d'Aquitaine (INCIA), Centre National de la Recherche Scientifique (CNRS), Unité Mixte de Recherche (UMR) 5287, University of Bordeaux, Bordeaux, France

**Keywords:** caudate nuclei, alcohol use disorder, relapsers, abstainers, structural magnetic resonance imaging, longitudinal analysis

## Abstract

**Background:**

Whether alteration in regional brain volumes can be detected in Type A alcoholics both at baseline and after a long follow-up remains to be confirmed. Therefore, we examined volume alterations at baseline, and longitudinal changes in a small follow-up subsample.

**Methods:**

In total of 26 patients and 24 healthy controls were assessed at baseline using magnetic resonance imaging and voxel-based morphometry, among which 17 patients and 6 controls were re-evaluated 7 years later. At baseline, regional cerebral volumes of patients were compared to controls. At follow-up, three groups were compared: abstainers (*n* = 11, more than 2 years of abstinence), relapsers (*n* = 6, <2 years of abstinence), and controls (*n* = 6).

**Results:**

The cross-sectional analyses detected, at both times, higher caudate nuclei volumes bilaterally in relapsers compared to abstainers. In abstainers, the longitudinal analysis indicated recovery of normal gray matter volumes in the middle and inferior frontal gyrus, and in the middle cingulate, while white matter volumes recovery was detected in the corpus callosum and in anterior and superior white matter specific regions.

**Conclusions:**

Overall, the present investigation revealed larger caudate nuclei in the relapser AUD patient group both at baseline and at follow-up in the cross-sectional analyses. This finding suggest that a higher caudate volume could be a candidate risk factor of relapse. In patients with specific type A alcohol-dependence, we showed that long-term recovery in fronto-striato-limbic GM and WM volumes occurs during long-term abstinence. These results support the crucial role of frontal circuitry in AUD.

## 1. Introduction

Alcohol use disorder (AUD) causes extensive cortical and subcortical Gray Matter (GM) and White Matter (WM) brain damage (1–3), characterized by lower regional volumes. There might be a change in key-brain regions modulated by treatments available for AUD, as it is a multidimensional disorder which includes several subtypes with different neurobiological underpinnings (4, 5).

Previous studies have not exclusively included individuals without neurological complications (i.e., type A alcohol-dependent individuals). There are scarce studies examining AUD's patients with Type A alcohol-dependence, as designated by Chanraud et al. (6) with no neurological, somatic or psychiatric complications, and for whom the onset of dependence occurred late in life (6–8). Compared to 24 controls, Chanraud et al. (6) showed decreases in gray matter volumes that were detected bilaterally in 26 Type A alcohol-dependent individuals in the dorsolateral frontal cortex (up to 20% lower), and in the temporal cortex, insula, thalamus, and cerebellum. Decreases in white matter volumes were widespread, reaching 10% in the corpus callosum (6). Fein et al. analyzed 24 young to middle-aged treatment-naïve Type A alcohol-dependent individuals who showed reduced whole brain, prefrontal, and parietal cortical gray matter volumes compared to 17 controls. These structural brain changes were negatively associated with age and lifetime duration of alcohol use, which were highly intricated. Temporal cortex and white matter did not differ between the two groups (7). Finally, Pfefferbaum et al. showed that 16 Type A alcohol-dependent patients had a cortical gray matter loss over time in the prefrontal cortex and the anterior superior temporal lobe and enlarged ventricles, compared to 28 controls who drank low amounts of alcohol (8).

Several longitudinal MRI studies investigated the short-term reversibility (up to 24 months) of these structural alterations. They compared AUDs vs. controls as well as abstainers vs. relapsers after a period of abstinence ranging from 1 to 24 months (9–22) (see for review Supplementary Table 1). In patients maintaining abstinence for over 3 months, regional GM volumes partially recovered in the cingulate cortex, the orbito-frontal cortex and the insula (15). In patients maintaining abstinence over 8 months a regional GM volumes recovery was shown in the frontal and parietal regions after 1 month (14, 16).

Furthermore, there are scarce reports on structural predictors of relapse between relapsers and abstainers. These studies highlight initial hypovolumetry, in the fronto-ponto-cerebellar and mesocorticolimbic regions (12, 14), in the bilateral frontal cortex (21), the frontal cortical thickness (16), as well as in the right orbito-frontal cortex, medial prefrontal cortex, and right anterior cingulate cortex regions (17), the amygdala (23) and the striatum and the thalamus (24).

However, the follow-up duration in all these longitudinal studies varied from 1 month to 2 years.

Thus, to our knowledge, no studies have investigated the brain structure damage in AUD between abstainers and relapsers after more than 24 months.

Besides most of the longitudinal studies conducted evaluated AUD patients with somatic and psychiatric comorbidities (12–16, 20, 21, 25) (see Supplementary Table 1). Few studies (11, 15, 17, 26) have explored uncomplicated AUD men and women, i.e., Type A, as designated by Babor et al. (27).

Therefore, our study aimed to detect whether there are brain damage differences beyond 24 months between relapsers and abstainers, particularly in Type A AUD patients, and identify regional volumes as potential predictors of outcome at baseline, or as predictors of reversibility at follow-up.

Therefore, we first compared WM and GM volumetry between all healthy controls and AUD patients at baseline.

The groups were formed according to the maintenance or not of abstinence at 7 years of follow-up; afterwards, at follow-up, we compared relapsers, abstainers, and controls groups.

Secondly, we aimed to investigate the long-term changes in regional volumes at follow-up, by comparing cross-sectionally and longitudinally the followed-up subgroups of abstainers, relapsers, and controls.

In line with the literature, we hypothesized that long-term abstinence would lead to, at least partial, recovery of the prefrontal cortex, cingulate cortex, and WM volume reductions.

## 2. Materials and methods

### 2.1. Participants

At baseline (BL) twenty-nine AUD patients detoxified for at least 3 weeks (mean age 47.4 ± 7.7 years), and meeting DSM-IV criteria for alcohol-dependence were recruited from consecutive admission to addiction disorders wards of addiction departments at Paul Brousse and Emile Roux Hospitals (AP-HP).

Twenty-nine healthy controls with neither past nor current substance abuse, matched to AUD patients for age, sex, Body Mass Index (BMI) and education were recruited from the neighboring community. Body Mass Index (BMI; kg/m^2^) was calculated as the ratio of patient collected data on weight and height and is defined as the weight divided by the square of the body height. Because some studies have reported sex-differences regarding alcohol-dependence (28), we chose to include only men in our study, in order to limit the impact of gender and heterogeneity in our limited sample size.

At baseline (BL), twenty-nine patients and twenty-nine healthy controls were recruited.

All patients and controls were males, Caucasian and right-handed as determined by the Annett Hand Preference Questionnaire (29).

Finally, due to motion artifacts and other technical difficulties, 3 AUD and 5 controls were excluded. Thus, 26 AUD and 24 controls were finally included in analyses at baseline [see Chanraud et al. (6) and the flow chart in Supplementary Figure 1].

The inclusion criteria for the control group were a consumption of less than two standard units of alcohol per week (20 g) during the previous year and a score of ≤ 5 on the Alcohol Use Disorders Identification Test (AUDIT) (30).

Exclusion criteria for both groups included being under 25 or over 65 years of age, in order to avoid age-related increased brain vulnerability to alcohol abuse (31–33). Other exclusion criteria were left- handedness, non-fluency in French, history of substance abuse or dependence other than caffeine and tobacco, sedative treatment for at least 1 week, axis I disorder (particularly mood and/or anxiety disorders, psychosis), high scores (>5) on the Hamilton Anxiety and Hamilton Depression Rating Scale (HARS and HDRS) (34, 35), malnutrition, hepatic pathology revealed by a ratio of liver enzymes aspartate aminotransferase/alanine aminotransferase (AST/ALT) greater than 2 (36), neurological and somatic diseases including a history of head injury with loss of consciousness, stroke, or other major brain abnormalities observed on MRI scans.

The characteristics of the participants' groups are provided in Table 1. The Bicêtre Ethics Committee (CPP-IDF 7) approved the study protocol. All participants received verbal and written protocol information, signed a consent form and received monetary compensation for their participation.

**Table 1:** Characteristics of the three participant groups and whole brain volumes at baseline and follow-up.

**At baseline**						**At follow-up**				
	**Relapsers (*****n** =* **6)**	**Abstainers (*****n** =* **11)**	**Controls (*****n** =* **24)**	**Kruskal-Wallis** **/Wilcoxon**		**Relapsers (*****n** =* **6)**	**Abstainers (*****n** =* **11)**	**Controls (*****n** =* **6)**	**Kruskal-Wallis /Wilcoxon**	
	**Means (SD)**	**Means (SD)**	**Means (SD)**	**Chi2/Z**	**P-value**	**Means (SD)**	**Means (SD)**	**Means (SD)**	**Chi2/Z**	**P-value**
Age (years)	44.17 (6.1)	47.73 (7.52)	44.25 (7.4)	2.09	0.35	50.83 (5.52)	54.91 (5.8)	54.33 (4.41)	1.39	0.5
Education (years)	13.16 (3.54)	12 (2.19)	13.54 (3.25)	1.45	0.48	13.16 (3.54)	12 (2.19)	14.16 (2.56)	2.10	0.34
BMI	22.16 (1.48)	23.94 (3.44)	24.66 (3.1)	3.81	0.15	23.31 (1.91)	24.74 (3.57)	24.37 (3.57)	1.03	0.59
Age of first drinking (years)	18.83 (5.53)	22.18 (10.07)	/	0.75	0.38					
Age of alcohol dependence onset (years)	41.75 (7.82)	49.5 (8.14)	/	2.28	0.13					
Total years of alcohol dependence	10.08 (7.26)	6.9 (5.56)		0.65	0.42	14.94 (7.88)	7.15 (5.13)		0.04	0.84
Alcohol family history (yes/no)	2y/4n	9y/2n		Fisher test	0.11					
Consumption (SDU)	27.50 (20.57)	32.91 (17.54)		0.65	0.42	20 (16.91)	5.18 (17.18)		11.76	**0.0006**
Prior detoxification treatments No	0.5 (0.54)	1 (1)		0.94	0.33	3.16 (1.47)	1.36 (1.63)		4.44	**0.04**
Length of abstinence (years)	0.43 (0.45)	1.18 (2.58)		0.45	0.5	0.36 (0.77)	6.02 (0.87)		11.19	**< 0.001**
Pack-years of active smoking	32.25 (18.30)	28.29 (19.31)	3.04 (8.34)	22.77	**< 0.0001**	39.83 (19.36)	31.80 (20.16)	2, 62 (6,41)	10.08	**0.006**
AST (U/l)	33.83 (20.97)	24.27 (6.77)		0.16	0.68	21.16 (4.11)	19.91 (6.36)		1.02	0.31
ALT (U/l)	31.66 (19.83)	23.81 (11.34)		1.12	0.29	19.66 (9.43)	24.63 (19.96)		0.16	0.68
Gamma GT	71.16 (95.41)	39.63 (43.81)		1.58	0.21	39.5(79.63)	29.82 (29.4)		3.13	0.07
HDRS	1 (1.55)	0.73 (1.01)		6.18	0.01	1 (0.83)	0.73 (1.01)		0.08	0.77
HARS	1.83 (1.17)	2.09 (1.51)		0.02	0.87	1.5 (2.35)	1.09 (1.04)		0.04	0.83
AUDIT	31 (4.98)	35.09 (4.15)		2.2	0.14	13.5 (15.1)	/			-
MMSE	28.16 (3.6)	29.63 (0.67)	29 (1.21)	3.1	0.21	29.17 (0.98)	29.36 (0.81)	29.16 (1.6)	0.27	0.87
SAS-SR	2.80 (0.46)	2.47 (0.52)		1.71	0.19					
CSF volume	367.67 (40.42)	376 (65.51)	306.7 (44.68)	12.1	**0.002**	426 (65.76)	380, 18 (61.1)	313 (35.83)	7.05	**0.03**
GM volumes	652 (37.9)	617.27 (52.22)	653.1 (42.1)	2.57	0.09	631 (34.1)	616, 73 (58.4)	611.16 (35.7)	0.39	0.82
WM volume	532.33 (45.46)	521.27 (39.37)	543 (50.68)	3.72	0.15	505.67 (49.5)	519, 09 (44.47)	506.16 (36.36)	0.20	0.9
TIV	1,551.8 (74.1)	1,514.9 (123.1)	1,502.6 (95.7)	1.4	0.5	1,562.8 (77.1)	1,516 (121, 84)	1,430.8 (97.5)	5.43	0.07

R, relapsers; A, abstainers; C, controls. BMI, body mass index; alcohol consumption in Standard Drinking Unit. Drinking Unit. Unity Laboratory norms. AST, aspartate aminotransferase; ALT, alanine aminotransferase; GGT, gamma-glutamyl-transferase; Tests: AUDIT, Alcohol Use Disorders Identification Test (range 0–40); MMSE, Mini-Mental State Examination; SAS-SR, Social Adjustment Scale Self Report; TIV, Total intracranial volume; GM, Gray Matter; WM, White Matter; CSF, Cerebrospinal fluid. Between-group comparisons were performed using Kruskal-Wallis and Wilcoxon tests. Bold P-values indicate a significant difference between groups (*p* < 0.05).

### 2.2. Clinical assessment

Trained psychiatrists (CM, EA, HJA, and JLM) performed a clinical evaluation of all the participants, examined their medical records and biological data at BL and follow up (FU). The presence of an axis I disorder (particularly mood and/or anxiety disorders, psychosis) was evaluated by a clinical interview. Trained psychiatrists (CM, EA, HJA, and JLM) interviewed and clinically evaluated patients, as well as examined their medical records and biological data. The diagnosis was determined after the clinical interview, by consensus of at least two interviewers and according to DSM-IV criteria.

Alcohol-dependence was assessed using the AUDIT and nicotine dependence by the Fagerström test (FTND) (38). Social functioning was evaluated using the Social Adjustment Scale Self Report (SAS-SR) (39), a self-report questionnaire, that evaluates daily functioning, and includes questions on leisure and social activities, relationships, economic status, marital status, children and extended family. Intellectual deterioration was assessed by the Mini-Mental State Examination (MMSE) (40), intellectual efficiency was assessed by the Information Subtest of the Weschsler Adult Intelligence Scale III Third Revision (41), and anxiety and depression were assessed using the Hamilton Anxiety Rating Scale and Hamilton Depression Rating Scale respectively (34, 35).

Moreover, we asked participants to rate among their first- and second-degree family members, the number of problematic alcohol drinkers.

Biological blood tests were performed for all subjects on both BL and FU. On the day of testing, fasting blood samples were drawn to investigate the somatic complications of chronic alcoholism. The panel of tests included liver function tests: aspartate aminotransferase (AST), alanine aminotransferase (ALT), gamma-glutamyl-transferase (GGT), carbohydrate-deficient transferrin (CDT), bilirubin, hemoglobin, hematocrit and mean blood volume (MCV). Abstinence was ascertained by normal blood levels of carbohydrate-deficient transferrin (CDT), of gamma-glutamyl-transferase (GGT), and of mean blood volume (MCV).

At FU, the AUDIT and the alcohol consumption self-report since BL were used to retrospectively estimate the quantity and the frequency of their alcohol consumption. Participants were also asked to report the duration and number of relapses and related-detoxifications during the follow-up period (from 0 to 3 detoxifications). Furthermore, we verified these data by reviewing their medical records.

Sub-group's characteristics: sub-groups were formed according to patients self-reported alcohol consumption during the 2 years before follow-up evaluation and confirmed by medical reports and blood alcohol tests [normal blood levels of gamma-glutamyl-transferase (GGT), carbohydrate-deficient transferrin (CDT) and mean blood volume (MCV)].

Abstainers self-reported no alcohol consumption for at least 2 years at FU that was confirmed by available medical records and available laboratory indicators of alcohol consumption [e.g., gamma glutamyltransferase (GGT)], which were within normal limits at follow-up. Relapsers self-reported alcohol consumption in the 2 years before FU, and this was confirmed by available medical records.

The two-year threshold is in line with the literature on long-term abstinent alcoholics, which commonly uses a duration of abstinence of more than 18 months (18, 37, 42) or more than 2 years (11, 43, 44).

All controls remained abstinent: three did not drink at all, one drank one drink per week, one consumed 2 drinks by month and one consumed 2 drinks by year. At 6-year follow-up, all AUD patients and controls were called by phone. The average duration between the baseline and follow-up MRI acquisitions was 77 ± 5 months and ranged from 68 to 85 months. Among the initial twenty-six AUD patients, eighteen were still followed in detoxification centers. Of those lost to follow up, two died between BL and FU. An additional research subject was excluded because of an incidental leukemia diagnosis. Five patients were unreachable. Among the twenty-four control subjects, seventeen were unreachable. At FU, a technical problem was encountered during the MRI acquisition of one patient and one control subject.

Overall, 17 AUD (11 abstainers and 6 relapsers) patients and 6 controls were included in the final analyses.

### 2.3. Imaging methods

#### 2.3.1. Magnetic resonance imaging acquisition

MRI data was acquired at BL and at FU on the same Signa 1.5 Tesla Whole Body system from General Electrics (Milwaukee, Wisconsin) at SHFJ (CEA, Orsay, France), with a standard 3D T1-weighted inversion recovery fast-spoiled gradient-recalled sequence with identical parameters: axial orientation, matrix = 256 × 192 interpolated to 256 × 256, 124 slice locations, 0.9375 × 0.9375 mm^2^ in-plane resolution, slice thickness = 1.3 mm, TE = 2 ms, TR = 10 ms, TI = 600 ms, flip angle = 10°, and read bandwidth = 12.5 kHz.

#### 2.3.2. Magnetic resonance image preprocessing

Spatial normalization and tissue segmentation in gray and white matter probability maps were performed for all images using the Cat12 toolbox (http://www.neuro.uni-jena.de/cat/), in SPM12 (Statistical Parametric Mapping, https://www.fil.ion.ucl.ac.uk/spm/) implemented in Matlab (https://fr.mathworks.com/help/matlab/ref/edit.html). Gray and white matter segmented images were modulated to compensate for deformations and finally smoothed with a 8-mm FWHM Gaussian filter. Total intracranial volume (TIV) was also estimated using the Cat12 toolbox. Visual quality control was performed for each raw image by one author (RM) and verified by another (CM). Cat12 quality rating was examined, and all preprocessed images were used.

Thus, participants underwent brain scanning both at BL and FU, using the same scanner, head coil, and volumetric MRI sequence parameters.

### 2.4. Statistical analyses

#### 2.4.1. Sociodemographic, clinical, and biological analyses

At BL and at FU, socio demographic, clinical and biological data, brain volumes of the three groups (relapsers, abstainers and controls) were compared, with the Jmp 14 software (https://www.jmp.com/fr_fr/home.html) using non-parametric tests such as Wilcoxon and Kruskal-Wallis.

All neuroimaging analyses were performed with SPM12, in whole brain. All scans were free from abnormalities.

#### 2.4.2. Cross-sectional voxel-based morphometry analyses

Cross-sectional voxel-based morphometry (VBM) analyses were performed on whole-brain GM and WM images (45) at both BL and FU.

A one-way ANOVA model in SPM was used, with group (relapsers, abstainers, controls) as the between-subject factor and age, years of education and TIV as confounding covariates.

At baseline, analyses were performed with all included controls (*n* = 24), and all AUD patients. Thereafter, we compared the baseline groups from participants included at both times (abstainers *n* = 11, relapsers *n* = 6, controls *n* = 6). At FU, analyses were conducted with all subjects included at both times (abstainers *n* = 11, relapsers *n* = 6, controls *n* = 6).

Statistical height threshold was set at *p* < 0.001 uncorrected, and extent threshold at *p* < 0.05 uncorrected (k = 300 voxels) (46). The get_totals SPM function was used to extract volumes from all significant clusters at BL (http://www0.cs.ucl.ac.uk/staff/g.ridgway/vbm/get_totals.m.).

#### 2.4.3. Longitudinal analyses

Longitudinal analyses were conducted using a flexible factorial design (one-way ANOVA for repeated measures) with time (BL and FU) as within-subject factor and group (relapsers, abstainers, controls) as between-subject factor.

As tobacco consumption may have confounding effects (20, 47), we conducted supplementary analyses with the number of cigarette packs smoked per year, entered as covariate.

In the longitudinal analyses, the height threshold was set at *p* < 0.001 uncorrected and the extent threshold at *p* < 0.05 uncorrected (respectively k = 150 voxels and k = 210 voxels for the GM and the WM analysis).

We used the AAL atlas (48) within the xjview toolbox (https://www.alivelearn.net/xjview/) and the JHU toolbox in MRIcron software to locate regions in all VBM analyses (49).

## 3. Results

### 3.1. Participant's characteristics

The three participant groups did not significantly differ in most socio-demographic data, biological variables, rating scales scores and whole brain tissues volumes except for CSF at BL and at FU, as described in Table 1. The duration of alcohol abstinence (*p* < 0.0001), alcohol consumption (*p* = 0.0006) and the number of withdrawals (*p* = 0.04) differed between the two subgroups of AUDs (abstainers vs. relapsers) at FU whereas no difference was found at BL. In addition, AUD patients smoked more than controls at both time points (BL: RvC Z = 4.08; *p* < 0.0001; AvC Z = 3.23; *p* < 0.0001; RvA Z = 0.15; *p* = 0.88; FU: RvC Z = 2.9; *p* = 0.004; AvC Z=2.65; *p* = 0.008; RvA Z=0.45; *p* = 0.65). All patients had good social functioning based on the SAS-SR scale: SAS-SR scale AUD subgroups scores were not different at BL (Table 1).

Thus, AUD patients were split into two groups: relapsers, who had been abstinent for <2 years (*n* = 6; mean 0.36 ± 0.77 years), and abstainers, whose duration of abstinence was >2 years (*n* = 11; 6.02 ± 0.87 years).

It is worth noting that no significant difference in education level, age, or BMI was found between the unreachable (*n* = 6) and the reachable (*n* = 17) controls (Supplementary Table 2).

### 3.2. MRI results

#### 3.2.1. Cross-sectional GM analyses at baseline

At BL, the comparison between controls and all AUD patients (controls *n* = 24; AUDs *n* = 17) revealed significant regional gray matter volume reductions in AUDs in bilateral hippocampus and para-hippocampus, left amygdala, bilateral medial frontal, right precentral, left temporal middle gyri and right thalamus (Supplementary Table 3). No larger GM volumes were found in AUD patients compared to controls.

**Figure 1:**
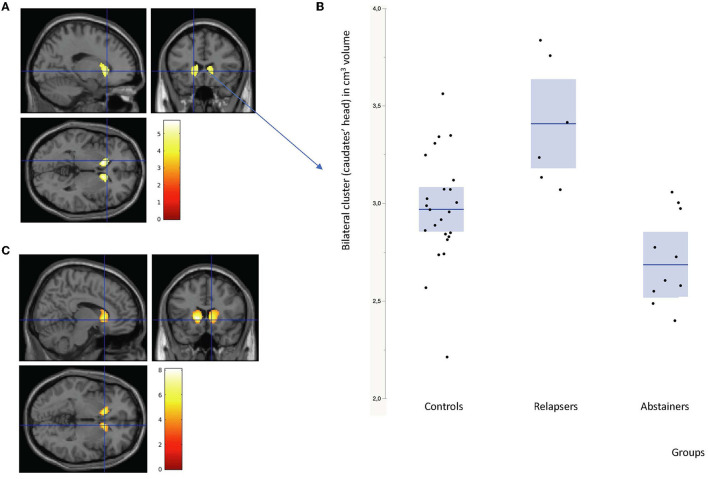
**(A)** Gray matter volumes: cross-sectional bilateral caudate nuclei increases in relapsers compared to abstainers Alcohol Use Disorders (AUD) patients, at baseline (height and extent threshold *p* < 0.05 FWE corrected). **(B)** Individual plots: bilateral heads of the caudate nuclei cluster volumes (in cm^3^) at baseline in controls on the left, relapsers on the middle and abstainers on the right. **(C)** Idem at follow-up (height threshold *p* < 0.001 uncorrected and extent threshold *p* < 0.05 FWE corrected).

The cross-sectional BL differences between subgroups (controls *n* = 24; relapsers *n* = 6; abstainers *n* = 11) with voxel-wise two-sample t-tests showed that the relapsers had a higher volume than the abstainers in the head of the caudate nucleus (CN) bilaterally (PFWE-corrected <0.05 at cluster and peak levels) (Table 2, Figure 1A). Individual plots of the bilateral heads of the caudate nuclei cluster volumes (in cm^3^), at baseline, in controls, relapsers and abstainers are represented in Figure 1B to illustrate this result. No significant difference was found among relapsers < abstainer's contrast.

**Table 2:** Gray Matter (GM) cross-sectional analyses: comparisons between relapsers and abstainers: Relapsers > Abstainers.

	**Regions**	**Cluster level**		**Peak level**		**MNI coordinates**		
		**k**	**p (FWE)-corr**	**t**	**p (FWE)-corr**	**x**	**y**	**z**
**Baseline**	R Caudate Head	1,091	0.007	6.21	0.01	10	18	4
	L Caudate Head	899	0.016	5.58	0.049	−12	16	0
**Follow-up**	L Caudate Head	987	0.011	8.09	0.009	−14	16	2
	R Caudate Head	1,055	0.008	6.09	0.161	10	16	0

P(FWE)-corr = P Family-Wise Error corrected; BA, Brodmann's area; L, left; R, right. No significant difference for WM cross-sectional relapsers vs. abstainers comparisons, at baseline and at follow-up.

With respect to controls, the relapsers only had a higher volume in the right caudate head (*p* < 0.001 uncorrected at peak level) (Supplementary Table 3).

Compared to controls, the abstainers had lower volumes in the right precentral gyrus, left hippocampus, left medial frontal gyrus, right para-hippocampus and bilateral thalamus (*p* < 0, 001 uncorrected at cluster and peak level) (Supplementary Table 3).

#### 3.2.2. Cross-sectional GM analyses at follow-up

At FU, the comparison between the reassessed controls and all AUD patients (AUDs *n* = 17; controls *n* = 6) revealed no significant regional GM volume reduction in all AUDs vs. controls contrasts.

The cross-sectional GM differences between subgroups (relapsers *n* = 6, abstainers *n* = 11, controls *n* = 6) with voxel-wise two-sample *t*-tests still showed higher volumes in the head of the CN bilaterally in relapsers compared to abstainers (see Table 2, Figure 1C). No significant difference was found in the relapsers < abstainers' comparison.

No significant GM volume difference was found between controls and both patient sub-groups (Supplementary Table 3).

#### 3.2.3. Cross-sectional WM analyses at baseline

At BL, the comparison between controls and all AUD patients (controls *n* = 24; AUDs *n* = 17) revealed widespread reductions of the regional WM volume in patients in the midbrain, left cerebral peduncle, right retrolenticular part of the internal capsule, superior and inferior longitudinal and inferior fronto-occipital fasciculi, right superior corona radiata and left corpus callosum. Reductions were also detected in the bilateral parietal and left middle occipital, superior temporal, cingulate, middle frontal, left medial and left superior frontal (Supplementary Table 3). No significant WM volume reduction was found in controls compared to AUD patients.

The cross-sectional BL comparisons between subgroups for volumes of WM (controls *n* = 24; relapsers *n* = 6; abstainers *n* = 11) showed no difference between relapsers and abstainers. However, compared to controls, relapsers had significant WM reduction adjacent to the bilateral thalamus, lingual, inferior frontal, and inferior parietal regions, as well as in the left cerebral peduncle, midbrain, and right superior longitudinal fasciculus (Supplementary Table 3). Also, compared to controls, abstainers had lower WM volume in the superior longitudinal fasciculus, left superior corona radiata, left anterior limb of internal capsule (ALIC), right external capsule, sagittal stratum and regions adjacent to the left putamen and bilateral frontal regions. No WM volume reduction was found in controls compared to abstainers (Supplementary Table 3).

#### 3.2.4. Cross-sectional WM analyses at follow-up

At FU, the comparison between the reachable controls and all AUD patients (controls *n* = 6; AUDs *n* = 17) revealed no significant regional WM volume differences, and none were found between subgroups (Supplementary Table 3).

The cross-sectional analysis results are maintained after control by tobacco consumption (pack/year).

#### 3.2.5. CSF at both BL and FU

AUD patients had significantly more CSF than controls at both BL and FU (BL: RvC Z = 2.70; *p* = 0.007; AvC Z = 2.79; *p* = 0.005; RvA Z = 0.45; *p* = 0.65; FU: RvC Z = 2.80; *p* = 0.005; AvC Z = 1.66; *p* < 0.1; RvA Z = 0.75; *p* = 0.45).

#### 3.2.6. Longitudinal gray matter analysis

**Figure 2:**
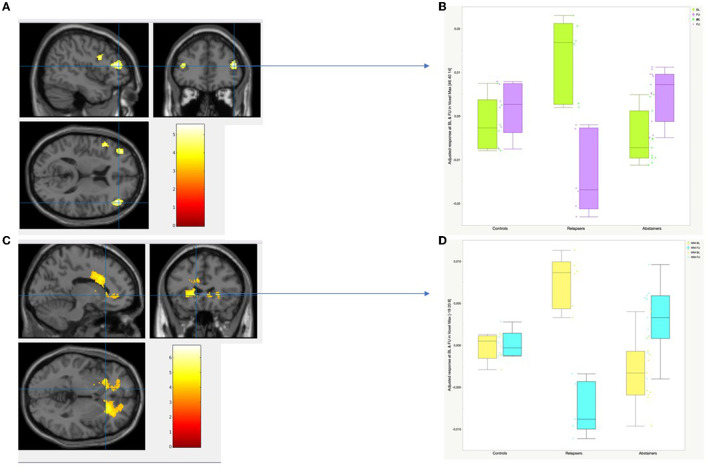
Longitudinal gray matter and white matter analysis: Time by Group Interaction. **(A)** 2D views of GM regions showing significant time x group interaction. The height threshold was set at *p* < 0.001 uncorrected and the extent threshold at *p* < 0.05 uncorrected. **(B)** Individual plots and adjusted voxel values at baseline (boxplots in green) and at follow-up (boxplots in purple) in control, relapser and abstainer groups at the highest peak-voxel detected by the Time by Group Interaction in GM (right middle frontal gyrus, BA 46: MNI coordinates: x = 46, y = 40, Z = 14). **(C)** 2D views of WM regions showing significant time x group interaction. The height threshold was set at *p* < 0.001 uncorrected and the extent threshold at *p* < 0.05 uncorrected. **(D)** Individual plots and adjusted voxel values at baseline (boxplots in yellow) and at follow- up (boxplots in blue) in control, relapser and abstainer groups at the highest peak-voxel detected by the Time by Group Interaction in WM (Anterior Limb of Internal Capsule: MNI coordinates: x = −18 y = 20, Z = 8).

For relapsers vs. abstainers, significant time (BL and FU) x group interactions were found in the frontal cortex bilaterally: middle frontal gyrus (BA 9, BA 10, and BA 46), inferior frontal gyrus, pars opercularis (BA 44), and the left precuneus. Other significant clusters include the bilateral middle cingulate (BA 24 and BA 32) (see Table 3, Figure 2A).

**Table 3:** Grey Matter (GM) and White Matter (WM) longitudinal analyses: comparisons between relapsers and abstainers.

**Interaction between times (BL–FU) and groups (abstainers vs. relapsers): GM longitudinal comparison**								
**Regions**	**BA**	**Cluster level**		**Peak level**		**MNI coordinates**		
		**k**	**p uncorr**.	**t**	**p uncorr**	**x**	**y**	**z**
R middle frontal gyrus	46	448	0.002^*^	5.55	1.43 × 10^−5^	46	40	14
L middle frontal gyrus	10	194	0.031^*^	5.01	4.54 × 10^−5^	−39	44	14
L inferior frontal gyrus, opercular part	44	166	0.044	5.01	4.58 × 10^−5^	−52	20	12
L precuneus/cingulate gyrus		437	0.003^*^	4.96	5 × 10^−5^	−4	−56	27
L precuneus/parietal lobe				4.78	7.41 × 10^−5^	−6	−64	33
R inferior frontal gyrus, opercular part	44	227	0.021	4.89	5.9 × 10^−5^	46	10	28
R middle frontal gyrus	9			4.52	1.31 × 10^−4^	42	3	38
L middle cingulate/supplementary motor aera	32	256	0.016	4.44	1.58 × 10^−4^	−4	9	45
L middle cingulate	24			4.34	1.99 × 10^−4^	−2	3	44
R middle cingulate	32			4.32	2.06 × 10^−4^	8	3	46
L middle cingulate	24			4.10	3.22 × 10^−4^	−6	−6	39
**Interaction between times (BL – FU) and groups (relapsers** > **abstainers): GM longitudinal comparison: no significative difference**.							
**Interaction between times (BL–FU) and groups (abstainers vs. relapsers): WM longitudinal comparison**.							
**Regions**	**Cluster level**		**Peak level**		**MNI coordinates**		
	**k**	**p uncorr**	**t**	**p uncorr**.	**x**	**y**	**z**
L anterior limb of internal capsule/caudate nuclei	2,511	2.52 × 10^−7^^*^	6.81	1.12 × 10^−5^^*^	−18	20	8
Body of corpus callosum/sub gyral frontal lobe			5.33	2.27 × 10^−5^	−16	12	26
L cingulum			4, 92	5.54 × 10^−5^	−8	21	27
L anterior corona radiata			4, 84	6.6 × 10^−5^	−28	22	6
L external capsule			4, 53	1.30 × 10^−4^	−28	16	6
R anterior corona radiata	1,173	1.06 × 10^−4^^*^	5.33	2.28 × 10^−5^	20	30	0
Genu of corpus callosum			4, 39	1.76 × 10^−4^	12	26	0
L inferior frontal gyrus, opercular part	215	0.05	5.22	3.23 × 10^−5^	−48	8	8
WM near R median cingulate gyrus	214	0.05	4.71	3.22 × 10^−4^	9	−42	33
WM near R posterior cingulate gyrus			3.37	3.7 × 10^−4^	8	−42	28
Splenium of corpus callosum			3, 28	5.24 × 10^−4^	4	−36	16
L inferior longitudinal fasciculus and inferior fronto occipital fasciculus	437	0.009^*^	4.64	1.02 × 10^−4^	−44	−20	−14
L superior longitudinal fasciculus/sub-gyral frontal lobe	280	0.03	4.56	1.22 × 10^−4^	−32	0	28

* Height or extend threshold <0.05 FWE corrected. The height threshold was set at *p* < 0.001 uncorrected and the extent threshold at *p* < 0.05 uncorrected; L, left; R, right. Interaction between times (BL–FU) and groups (relapsers > abstainers): WM longitudinal comparison: no significative difference.

GM volume decreased in relapsers over the course of 7 years in all listed regions, while abstainers displayed GM volume increase. An example is given for the middle frontal region in Figure 2B.

For controls vs. relapsers, significant time (BL and FU) x group interactions were found in the pars triangularis of the inferior frontal gyrus bilaterally. *Post-hoc* analyses indicated that relapsers had lost GM volume in this region while it had increased in controls over time.

For controls vs. abstainers, no significant time (BL, FU) x group interactions were found. Supplementary Table 4 reports all time (BL and FU) x group (relapsers, abstainers, and controls) GM volume interactions.

#### 3.2.7. Longitudinal white matter analysis

For the relapsers vs. abstainers, significant time (BL, FU) x group interactions were found in all parts of the corpus callosum. The same interactions were found in left anterior limb of internal capsule, bilateral corona radiata, external capsule, in the regions adjacent to caudate nuclei and the cingulate gyrus (all cluster-level PFWE-corrected ≤ 0.05). Interactions were also found in the left inferior and superior longitudinal fasciculi, inferior fronto-occipital fasciculus, and regions adjacent to the inferior frontal gyrus, pars opercularis (see Table 3, Figure 2C).

For all listed regions, WM volume decreased in relapsers and increased in abstainers over the course of 7 years. An example is given for the left ALIC region in Figure 2D.

For the controls vs. relapsers, significant time (BL, FU) x group interactions were found in regions adjacent to the insula, as well as in the right external capsule, right anterior corona radiata and right ALIC (see Supplementary Table 4).

For the controls vs. abstainers, no significant time (BL, FU) x group interactions were found. The Supplementary Table 4 reports the time (baseline and follow-up) × group (relapsers, abstainers, and controls) WM volume interactions.

## 4. Discussion

Regional tissue volume was different during long-term (7-year) recovery in a Type A alcohol-dependent sample compared with a control group. Yet, the final numbers of participants in each group (primarily abstainers and relapsers and controls) were small and due to the small patient sample, the present findings have to be considered as exploratory. Future studies are therefore necessary to confirm these findings in larger groups. It is however noteworthy that the finding of larger caudate nuclei appears to dissociate specifically relapsers from abstainers both at BL and FU. This main finding is supported by other ones in the present study, which are in agreement with previous literature concerning shorter follow-ups (generally up to 2 years).

In line with the literature, at BL, AUD patients relative to healthy controls showed smaller gray matter volume in limbic structures (hippocampus, para-hippocampus, amygdala) as well as the medial frontal and temporal regions, the precentral gyrus, and thalamus (12, 18, 23, 47, 48). In this same comparison, a significant decrease in the volume of WM is extensive in the brainstem, in the cerebral peduncle, in the anterior regions (the right internal capsule, the superior, and anterior right corona radiata), in the cingulum middle bilaterally and the right inferior and superior longitudinal bundle, in the right fronto-occipital inferior bundle, and in the commissural fibers of the corpus callosum (genu). Volume reductions were also detected in the right superior temporal WM, the bilateral sub gyral and middle frontal WM, in the left median and superior frontal WM, the right parietal WM and in the left occipital WM (49–52). These decreases in volumes of both WM and GM correspond to the parallel increase in the volume of CSF (12, 48, 53, 54) and further confirm our results.

Both at BL and at FU, relapsers had larger heads of caudate nuclei (CN) than abstainers bilaterally.

Longitudinal analyses showed recovery of normal GM volumes in the bilateral middle and inferior frontal, left precuneus and the bilateral mid-cingulate after long-term abstinence. Findings pointed to potential recovery of WM volume in adjacent regions, as well as in commissural tracts, the corona radiata bilaterally, left ALIC, the external capsule and the left superior and left inferior longitudinal fasciculus and the inferior fronto occipital fasciculus.

### 4.1. Cross-sectional gray matter analysis: The caudate nuclei

The finding of larger heads of CN in relapsers compared to abstainers was bilateral and symmetrical at both BL and FU, indicative of its robustness. The longitudinal analysis did not detect any significant change in this region, confirming the stability over time of the larger CN volumes in relapsers. This suggests that the pre-existing CN volume difference at baseline might be associated with a risk of relapse and thus could be a candidate vulnerability factor. It is strikingly consistent with a recent IMAGEN consortium study reporting higher GM volume in bilateral CN at age 14, as a structural brain predictor of a larger increase in alcohol use scores over 5 years, between age 14 and 19 (55). While both studies used voxel-wise analyses methods over the whole-brain volume (Supplementary Figure 2), the present findings in AUD relapsers confirm the location of their CN findings. This is consistent with the hypothesis that larger CN may indirectly denote vulnerability to poor alcohol use outcome.

Several reports below are of note to support this suggestion.

For instance, enlarged CN volume was reported in binge drinkers (56), in cocaine dependence (57, 58) and in methamphetamine dependence (59, 60). Moreover, only a few studies in AUD patients report a longitudinal exploration of the CN volume, notably in Type A alcohol-dependent subjects, and their follow-up durations were much shorter, ranging from 3 weeks to 18 months. Among eleven longitudinal and cross-sectional studies comparing abstinent AUD patients vs. controls, seven did not explore the caudate nuclei volumes (8, 19, 61–65) three did not find any significant difference (24, 66, 67) and one reported a reduction in CN volume (68). Two previous studies comparing AUD patients abstinent for 6 years to controls, but without any longitudinal design, and did not find any difference in CN volume either (42, 69) (see Supplementary Table 1 for a review).

Among twelve longitudinal studies comparing abstainers vs. relapsers, one reported a tendency toward CN volume increase in abstainers (12) at 7-months follow- up, one noted CN volume heterogeneity (20), and five did not find any differences in CN volume between groups (13, 14, 18, 20, 21).

The remaining studies did not explore potential differences in CN volume (9, 11, 15, 17, 22, 26).

Critically, most of these studies used data from patients with comorbidity (addictions and mental health disorders) (see Supplementary Table 1). This variety of subjects contrasts with the homogeneity of our own AUD patient sample, which might account for the detection of higher CN volume in relapsers. The filter used to smooth Jacobian maps could contribute to a difficulty in accurate detection of brain matter volume differences (12). Moreover, we can note other differences in the methodology used in the only longitudinal report over 18-months in abstainers vs. relapsers, which did not find any difference in CN volume, which included the manual delineation of brain regions, with no voxel-based analysis, and a mixed-gender sample (70).

The caudate nucleus mediates higher cognitive functions, including the executive functions and cognitive control (71–73), and is highly connected with the prefrontal cortex (74).

Moreover, the CN is implicated in the reward system (75, 76). The dorsal striatum, including CN and putamen, has been strongly linked to the development and expression of habituation behaviors (72, 77, 78). A link was made between enlarged striatal volumes and higher dopamine synthesis capacity, with an increase in dopamine level in the dorsal striatum, including the caudate and putamen (79, 80). In an fMRI study, when presented with alcohol-associated stimuli, dependent AUD patients showed hyper-activation of the caudate nuclei (81).

These data and the present exploratory results may suggest that individuals who recruit more often or more strongly motivational or reward circuits have larger CNs and are more likely to feel alcohol craving and thus relapse. Replication of our findings in a larger sample could allow further confirmation of this potential risk factor for alcohol consumption relapse. Therefore, supplementary investigations are needed to test the hypothesis of an enlargement of the CN, as an appetitive region, and a risk factor of relapse through automatic behavior.

### 4.2. Cortical gray matter longitudinal analysis

In line with the literature and with our hypotheses, abstainers compared to relapsers showed an increase in GM volume in a number of frontal regions, including the bilateral middle (BA 9, BA 10, and BA 46) and inferior (BA 44) frontal cortex, left precuneus and bilateral mid-cingulate (BA 24, 32). Consistently, previous longitudinal comparisons among abstainers and relapsers mentioned similar results, with a frontal GM volume increase already detected after 4 weeks of abstinence (22). After 3 months (15), then 8 months of abstinence (12), GM volume recovery was reported in the cingulate cortex. After 12 months of abstinence, a GM volume increase was also detected in various frontal regions in abstainers, in the superior frontal gyrus and orbitofrontal cortex (14–16), middle frontal cortex (16), middle and anterior frontal cortices (12), anterior mesial and prefrontal cortices (9), dorsolateral frontal cortex (13), and inferior frontal cortex (26).

We can also note that the consistency of the present findings with the previous literature supports that such volumetric changes can be detected using longitudinal voxel- and pair- wise methods in small and homogeneous groups of abstainers and relapsers followed-up during a longer time.

Herein, no longitudinal difference was detected between abstainers and controls in GM volume. This may indicate a recovery of the cortical GM volume. Previous longitudinal studies comparing AUD patients and controls found smaller volumes in the medial frontal and lateral prefrontal cortices (66). After 7 months of abstinence, a volume increase was reported in the dorsolateral and orbitofrontal cortices (65).

Overall, our results are mostly in line with the literature, showing general frontal hypovolumetry in relapsers compared to abstainers, the latter having possibly recovered GM volumes at long-term (7 years).

### 4.3. White matter longitudinal analysis

In line with the literature and with our hypotheses, relapsers compared to abstainers showed a widespread WM volume increase along with long-term abstinence in the cingulum, inferior frontal and temporal regions and adjacent to the bilateral CN. WM volume increase was also detected in the commissural tracts (genu, body and splenium of the corpus callosum), the corona radiata bilaterally, left ALIC, the external capsule and the left superior and left inferior longitudinal fasciculi, and finally the inferior fronto occipital fasciculus.

This is in line with previous longitudinal reports stating that relapsers compared to abstainers have smaller WM volume after 24 months (11), after 13 months (9) and after as early as 8 months of abstinence, in the brainstem, corpus callosum, cerebellum, bilateral temporal, anterior, and middle frontal WM connected to the bilateral orbitofrontal cortex (12), and in the WM in close proximity to the right frontal cortex and adjacent to anterior cingulate (14). Thus, WM volume starts to increase in a linear manner in AUD patients after at least 7.5 months of abstinence (20, 21) and the present results support that this effect remains on the long-term.

No significant difference in WM volume was found, after 7 6? years, between controls and abstainers, in line with reports of recovered WM assessed by Diffusion Tensor Imaging (20, 21) in abstainers, although with shorter abstinence duration.

On a speculative note, we provide evidence of volume recovery with abstinence in cortical regions and WM, while volumes in appetitive (sub-cortical CN) regions did not vary. Imbalance between the “appetitive” network including the CN, and the “executive” network including the cingulate and prefrontal cortex (82), might therefore lead to a failure to optimize the regulation of relevant functions (follow-up of recent actions, anticipation of results and action choice) that could *a fortiori* increase vulnerability to relapse.

### 4.4. Limitations

Due to our stringent exclusion criteria and the rigorous quality control processes, eligible patient profiles (only male Caucasian subjects, characterized by good social functioning, and preserved executive functions) were rare in a hospital setting. As we included subjects with Babor's Type A alcohol addiction, our results cannot be generalized to all AUD patients. Our sample was homogeneous but small, making our findings mostly exploratory and further studies are needed to extend our results to all alcohol-dependent patients.

Indeed, from the 26 patients and 24 controls recruited at baseline, we were only able to re-include 17 patients and 6 controls at follow-up. Most control participants were difficult to reach and were lost at follow-up due to the long duration of the study. This could explain that we did not find any difference between groups in the cross-sectional analysis, at follow-up.

As we already mentioned, many participants were lost at follow-up due to the long duration of the study but also to technical difficulties (cf Flow chart in Supplementary Figure 1). Consequently, our small sample limited our possibilities to highlight correlations between neuroimaging and neuropsychology.

A technical limitation in long-term longitudinal studies is linked to evolving MRI methods. At baseline, and then at follow-up, we had access to a 1.5 Tesla MRI, but, at follow-up, the acquisition settings had been slightly updated twice. However, the same machine was used for both evaluations. We could have used another MRI machine at high field strengths at follow-up, but this would have created another bias.

Further studies should continue to investigate other typologies of alcohol-dependence, such as Type B alcoholism, which is often associated with family history of alcoholism and related genetic data (*BDNF* gene). Some studies showed that among AUD patients and after 7 months of abstinence, BDNF gene Val/Val homozygotes displayed an increase in hippocampal volume compared to Val/Met heterozygotes (19). Another study showed a caudate nuclei volume decrease among Val/Val after 5 weeks of abstinence, but not among Val/Met (68). Mon et al. (68), showed that caudate nuclei volume recovery in abstinent Type A alcohol-dependent individuals was dependent on *BDNF* genotype. Indeed, among 41 middle-aged alcohol-dependent subjects (including 5 women), who started their heavy drinking around 27 years old and without biomedical or psychiatric disorders, Val/Val genotype patients had a caudate nuclei volume decrease after 5 weeks of abstinence, whereas Val/Met did not. The BDNF Val66Met (rs6265) polymorphism was significantly related to the recovery of regional GM tissue volumes within the first 5 weeks of sobriety, suggesting genetic influences on brain tissue changes during abstinence from alcohol in this Type A alcohol-dependent cohort. The BDNF is associated with neuronal survival, neuronal growth and synaptic plasticity in the adult brain (83). The allelic association of the A1 allele of the *DRD2* gene with alcohol-dependence was found in males but not in females. This discrepancy could be explained by the gender difference in dopamine D2-like receptor affinity and levels (84). Moreover, it has been shown that the presence of this A1 allele of the *DRD2* gene is correlated with a lower density of the D2 receptor in the striatum, including the caudate nucleus (85).

Overall, these results suggest that caudate volume in males with type A alcoholism could be associated with *BDNF* genotype. In our study, we did not perform genetic analyses due to our small sample and due to the scarcity of genetic assays.

## 5. Conclusions

The present findings raise the hypothesis of higher caudate GM volume to be a candidate risk factor of relapse. In patients with specific type A alcohol-dependence, we showed that long-term recovery in fronto-striato-limbic GM and WM volumes occurs during long-term abstinence. These results support the crucial role of frontal circuitry in AUD.

## Data availability statement

The original contributions presented in the study are included in the article/Supplementary material, further inquiries can be directed to the corresponding author.

## Ethics statement

The studies involving human participants were reviewed and approved by the Bicêtre Ethics Committee (CPP-IDF 7) had approved the study protocol. The patients/participants provided their written informed consent to participate in this study.

## Author contributions

J-LM obtained funding for the study. J-LM, AB, and CM designed the study and wrote the protocol. EA, J-LM, and CM conducted literature searches and analyses. SC, CM, and H-JA recruited participants. CM, EA, and J-LM conducted their clinical assessments. SC, CM, J-LM, and EA assisted the MR image acquisition in patients and controls. EA conceptualized and designed longitudinal analyses. CM and RM conducted cross-sectional and longitudinal image data processing as well as all statistical analyses. CM, EA, and J-LM interpreted the data, prepared the manuscript and wrote and edited the manuscript. RM, BR, AP, H-JA, AA, SC, and AB critically reviewed the manuscript for intellectual content and edited of final draft. All authors agree for all aspects of this work. All authors approved the final version of the manuscript for publication.
