# Supplementary material for: Caudate gray matter volumes and risk of relapse in Type A alcohol-dependent patients: A 7-year MRI follow-up study
Source: Front Psychiatry. 2023 Feb 15;14:1067326. doi: 10.3389/fpsyt.2023.1067326 (PMC9975333; doi:10.3389/fpsyt.2023.1067326)

**Table 1.** Subcortical neuroimaging studies review

**Table 2.** Controls participants reassessed at follow-up (N=6) did not differ from those lost at follow-up (N=17) regarding their demographic characteristics at baseline (age, body mass index, education), supporting their representativeness.

**Table 3.** Cross-sectional comparisons

1. At baseline MRI results of 41 subjects: Grey Matter (GM) VBM analyses.
2. At follow-up MRI results of 23 subjects: Grey Matter (GM) VBM analyses.
3. At baseline MRI results of 41subjects: White Matter (WM) VBM cross sectional analyses
4. At follow-up MRI results of 23 subjects: White Matter (WM) VBM analyses.

**Table 4.** Longitudinal analyses

**Figure 1.** Design of the longitudinal study: a 7-year follow-up

**Figure 2.** Whole brain analyses comparison between the present results and Kühn’s study.

**Table 1.** Subcortical neuroimaging studies review


**Table 2.** Controls participants reassessed at follow-up (N=6) did not differ from those lost at follow-up (N=17) regarding their demographic characteristics at baseline (age, body mass index, education), supporting their representativeness (Wilcoxon test).

|  |  | Mean | Standard Deviation | Z | p |  |
| --- | --- | --- | --- | --- | --- | --- |
| Education at BL (yrs) | Reassessed | 13.83 | 3.12 | 0.39 | 0.7 | NS |
|  | Dropped out | 13.47 | 3.55 |  |  |  |
| Age at BL (yrs) | Reassessed | 48 | 4 | 1.72 | 0.09 | NS |
|  | Dropped out | 43.29 | 8.15 |  |  |  |
| BMI at BL | Reassessed | 24.25 | 3.73 | 1.22 | 0.22 | NS |
|  | Dropped out | 26.3 | 4.76 |  |  |  |

**Table 3:** Cross-sectional comparisons

1. **At baseline MRI results of 41 subjects: Grey Matter (GM) VBM analyses.**

- **GM cross-sectional comparisons: Controls (N=24) > AUD (N=17)**

|  | **Regions** | **BA** | **Cluster level** | | **Peak level** | | **MNI coordinates** | | | |  |
| --- | --- | --- | --- | --- | --- | --- | --- | --- | --- | --- | --- |
|  |  |  | **k** | **p uncorr** | **t** | **p uncorr** | **x** | **y** | **z** |  |  |
|  | L hippocampus |  | 994 | 0.001 | 5.33 | **2.92.10^-6 *^** | -28 | -16 | -9 |  |  |
|  | L parahippocampus |  |  |  | 4.74 | 1.73. 10^-5^ | -20 | -34 | -8 |  |  |
|  | L amygdala |  |  |  | 4.01 | 1.50.10^-4^ | -20 | -10 | -10 |  |  |
|  | R parahippocampus |  | 2019 | 0.00002 | 5.23 | **4.10^-6 *^** | 24 | -24 | -18 |  |  |
|  | R hippocampus |  |  |  | 4.29 | 8.4.10-^6^ | 27 | -21 | -14 |  |  |
|  | R precentral | **6** | 727 | 0.004 | 5.13 | 5.4.10^-6^ | 52 | -4 | 38 |  |  |
|  | R precentral gyrus |  |  |  | 4.30 | 8,4.10^-6^ | 58 | -3 | 38 |  |  |
|  | L medial frontal gyrus, frontal sup medial | **6** | 314 | 0.04 | 4.95 | 9.3.10^-6^ | -2 | 44 | 42 |  |  |
|  | L medial frontal gyrus, frontal sup medial | **9** |  |  | 4.04 | 1.4.10^-4^ | -2 | 45 | 27 |  |  |
|  | L temporal middle | **22** | 443 | 0.02 | 4.47 | 3.91.10^-5^ | -57 | -44 | 6 |  |  |
|  | L temporal middle | **21** |  |  | 4.41 | 4.65.10^-5^ | -60 | -36 | 2 |  |  |
|  | R thalamus |  | 390 | 0.027 | 4.07 | 1.28.10^-4^ | 3 | -4 | 6 |  |  |
|  | R subthalamic nucleus |  |  |  | 3.92 | 1.97.10^-4^ | 4 | -14 | -4 |  |  |

***Height or extend threshold <0.05 FWE corrected.** The height threshold was set at p < 0.001 uncorrected and the extent threshold at p <0.05 uncorrected; BA=Brodmann’s area; L=left; R=right

- **No significant difference for GM cross-sectional comparisons : Controls < AUD**
- **GM cross-sectional comparisons : Relapsers > Controls**

| **Regions** |  | **Cluster level** | | **Peak level** | | **MNI coordinates** | | | |
| --- | --- | --- | --- | --- | --- | --- | --- | --- | --- |
|  |  | **k** | **p uncorr** | **t** | **p uncorr** | **x** | **y** | **z** |  |
| R Caudate head |  | 443 | 0.019 | 4,86 | 1.21.10^-5^ | 10 | 18 | 4 |  |

- **No significant difference for GM cross-sectional comparisons: Relapsers < Controls**
- **GM cross-sectional comparisons: Controls > Abstainers**

| **Regions** | **BA** | **Cluster level** | | **Peak level** | | **MNI coordinates** | | | |
| --- | --- | --- | --- | --- | --- | --- | --- | --- | --- |
|  |  | **k** | **p uncorr** | **t** | **p uncorr** | **x** | **y** | **z** |  |
| R precentral gyrus, precentral part | **6** | 1725 | **5.67.10^-5 *^** | 5.62 | **1.22.10^-6 *^** | 52 | -4 | 36 |  |
| R precentral gyrus, postcentral part | **3** |  |  | 4,17 | 9.6. 10^-5^ | 28 | -30 | 60 |  |
| L hippocampus |  | 537 | 0.01 | 5.14 | 5.21.10^-6^ | -28 | -15 | -9 |  |
| L medial frontal gyrus, frontal sup medial | **8** | 716 | 0.004 | 5.10 | 5,92.10^-6^ | -2 | 45 | 42 |  |
| L medial frontal gyrus, frontal sup medial | **9** |  |  | 4,89 | 1.12.10^-5^ | -2 | 45 | 27 |  |
| R parahippocampus | **35** | 314 | 0.04 | 4.56 | 3.02.10^-5^ | 24 | -22 | -20 |  |
| R thalamus |  | 552 | 0.01 | 4.17 | 9.54.10^-5^ | 8 | -9 | 2 |  |
| L thalamus |  |  |  | 3.58 | 5.08.10^-4^ | -4 | -9 | 8 |  |

*** Height or extent threshold at p<0.05 FWE corrected.** The height threshold was set at p < 0.001 uncorrected and the extent threshold at p <0.05 uncorrected; BA = Brodmann Area, R = right, L = Left.

| - **No significant difference for GM cross-sectional comparisons : Controls < Abstainers** |
| --- |

1. **At follow-up MRI results of 23 subjects: Grey Matter (GM) VBM analyses.**

- No significant difference for GM cross-sectional Controls > AUD and Controls < AUD comparisons
- No significant difference for GM cross-sectional Relapsers > Controls and Relapsers < Controls comparisons
- No significant difference for GM cross-sectional Controls > Abstainers and Controls < Abstainers comparisons

1. **At baseline MRI results of 41subjects: White Matter (WM) VBM cross sectional analyses.**

- **WM cross-sectional comparisons: Controls (N=24) > AUD (N=17)**

| **Regions** |  | **Cluster level** | | **Peak level** | | **MNI coordinates** | | | |
| --- | --- | --- | --- | --- | --- | --- | --- | --- | --- |
|  |  | **k** | **p uncorr** | **t** | **p uncorr** | **x** | **y** | **z** |  |
| L cerebral peduncle |  | 6707 | **1.50.10^-11^ *** | 5.52 | 1.66.10^-6^* | -20 | -20 | -10 |  |
| Midbrain |  |  |  | 5.38 | 2.5.10^-6^ | -18 | -20 | -3 |  |
| R WM near parahippocampus |  |  |  | 4.84 | 1.3.10^-5^ | 20 | -16 | -14 |  |
| R temporal superior |  |  |  | 4.7 | 1.97.10^-5^ | 45 | -26 | 2 |  |
| R retrolenticular part of internal capsule |  |  |  | 4.25 | 7.57.10^-5^ | 34 | -26 | 0 |  |
| R sagittal stratum include inferior longitudinal and inferior fronto-occipital fasciculus |  |  |  | 4.20 | 8.73.10^-5^ | 34 | -26 | -4 |  |
| L WM sub gyral frontal lobe |  | 6277 | **4.42.10^-11^ *** | 5.50 | 1.74.10^-6^* | -30 | -3 | 33 |  |
| L WM cingulum |  |  |  | 4.40 | 4.9.10^-5^ | -15 | -4 | 40 |  |
| L WM anterior cingulate |  |  |  | 4.28 | 6.94.10^-5^ | -15 | 30 | 26 |  |
| L superior corona radiata |  |  |  | 4.24 | 7.81.10^-5^ | -21 | -20 | 36 |  |
| L WM medial frontal |  |  |  | 4.14 | 1.02.10^-4^ | -21 | 34 | 21 |  |
| L Sub gyral parietal lobe |  |  |  | 4.08 | 1.23.10^-4^ | -27 | -34 | 44 |  |
| R WM sub gyral frontal lobe |  | 9410 | **2.73.10^-14^ *** | 5.38 | 2.52.10^-6^ | 27 | -22 | 50 |  |
| R superior longitudinal fasciculus |  |  |  | 4.69 | 2.05.10^-5^ | 30 | -2 | 26 |  |
| R sub gyral parietal lobe |  |  |  | 4.50 | 3.63.10^-5^ | 30 | -33 | 40 |  |
| R WM middle frontal |  |  |  | 3.85 | 2.37.10^-4^ | 26 | 48 | -2 |  |
| R superior corona radiata |  |  |  | 3.52 | 6.06.10^-4^ | 15 | 8 | 39 |  |
| R WM cingulum |  |  |  | 3.46 | 7.27.10^-4^ | 14 | -24 | 38 |  |
| L WM fusiform temporal lobe |  | 517 | 0.01 | 4.35 | 5.63.10^-5^ | -46 | -57 | -15 |  |
| L posterior thalamic radiation, include optic radiation |  |  |  | 4.32 | 6.08.10^-5^ | -38 | -56 | -2 |  |
| L WM middle occipital gyrus |  |  |  | 3.98 | 3.16.10^-4^ | -36 | -66 | 2 |  |
| L WM insula |  | 328 | 0.036 | 3.67 | 4.10^-4^ | -38 | -14 | -2 |  |
| L WM sub gyral frontal lobe |  | 644 | **0.005 *** | 4.13 | 1.06.10^-4^ | -20 | 32 | -10 |  |
| L WM anterior cingulate |  |  |  | 3.96 | 1.74.10^-4^ | -8 | 30 | -2 |  |
| L WM superior frontal |  |  |  | 3.93 | 1.93.10^-4^ | -22 | 50 | -4 |  |
| L WM middle frontal |  |  |  | 3.77 | 3.03.10^-4^ | -22 | 44 | -9 |  |
| L genu of corpus callosum |  |  |  | 3.65 | 4.29.10^-4^ | -14 | 27 | 0 |  |
| *** Height or extent threshold at p<.05 FWE corrected.** The height threshold was set at p < .001 uncorrected and the extent threshold at p <.05 uncorrected; R = right, L = Left | | | | | | | | | |

- **No significant difference for WM cross-sectional Controls < AUD comparisons**
- **No significant difference for WM cross-sectional Relapsers > Controls comparisons**
- **WM cross-sectional comparisons: Controls > Relapsers**

| **Regions** |  | **Cluster level** | | **Peak level** | | **MNI coordinates** | | | |
| --- | --- | --- | --- | --- | --- | --- | --- | --- | --- |
|  |  | **k** | **p uncorr** | **t** | **p uncorr** | **x** | **y** | **z** |  |
| L ventral diencephalon |  | 1688 | **0.00005 *** | 5.24 | 3.9.10^-6^ | -14 | -21 | 0 |  |
| L cerebral peduncle /L thalamus |  |  |  | 5.23 | **3.95.10^-6^ *** | -20 | -20 | -10 |  |
| R ventral diencephalon |  | 1536 | **0.00009 *** | 4.97 | **8.82.10^-6^ *** | 14 | -9 | -16 |  |
| Midbrain |  |  |  | 4.41 | 4.76.10^-5^ | 20 | -20 | -12 |  |
| R thalamus |  |  |  | 4.33 | 5.9.10^-5^ | 14 | -18 | -2 |  |
| L lingual /gyrus lingual |  | 353 | 0.03 | 3.58 | 5.16.10^-4^ | -20 | -66 | 0 |  |
| R precentral / Inferior frontal gyrus |  | 474 | 0.01 | 4.31 | 6.28.10^-5^ | 38 | -2 | 36 |  |
| R superior longitudinal fasciculus |  |  |  | 3.47 | 7.10^-4^ | 39 | -10 | 24 |  |
| R inferior parietal lobule |  | 547 | 0.009 | 4.28 | 8.87.10^-5^ | 38 | -40 | 40 |  |

- **WM cross-sectional comparisons: Controls > Abstainers**

| **Regions** |  | **Cluster level** | | **Peak level** | | **MNI coordinates** | | | |
| --- | --- | --- | --- | --- | --- | --- | --- | --- | --- |
| R sub gyral frontal lobe |  | 7570 | **1.85.10^-12^ *** | 5.80 | **7.05.10^-7^ *** | 27 | -22 | 48 |  |
| R superior longitudinal fasciculus |  |  |  | 4.48 | 3.82.10^-5^ | 30 | 4 | 21 |  |
| L WM near putamen |  | 1076 | **6.37.10^-4^ *** | 5.62 | **1.24.10^-4^ *** | -20 | 8 | -8 |  |
| L sub gyral frontal lobe |  |  |  | 3.68 | 3.94.10^-4^ | -20 | 30 | -12 |  |
| L frontal sup orb L |  |  |  | 3.52 | 6.02.10^-4^ | -15 | 22 | -15 |  |
| L superior corona radiata |  | 5199 | **7.37.10^-10 *^** | 4.89 | 4.51.10^-5^ | -30 | -3 | 32 |  |
| L anterior limb of internal capsule |  |  |  | 4.03 | 1.44.10^-4^ | -24 | 12 | 15 |  |
| R external capsule |  | 1582 | **7.39.10^-5 *^** | 4.42 | 4.59.10-5 | 34 | -15 | -8 |  |
| L sagittal stratum include inferior longitudinal fasciculus and inferior fronto occipital fasciculus |  | 507 | 1.10^-2^ | 4.15 | 1.01.10^-4^ | -38 | -20 | -10 |  |

*** Height or extent threshold at p<0.05 FWE corrected.** The height threshold was set at p < 0.001 uncorrected and the extent threshold at p <0.05 uncorrected; R = right, L = Left.

- **No significant difference for WM cross sectional Abstainers > Controls comparison**

1. **At follow-up MRI results of 23 subjects: White Matter (WM) VBM analyses.**

- **No significant difference for WM cross-sectional: Controls > AUD and Controls < AUD comparisons**
- **No significant difference for WM cross-sectional: Relapsers > Controls and Relapsers < Controls comparisons**
- **No significant difference for WM cross-sectional: Controls > Abstainers and Controls < Abstainers comparisons**

| \| **Table 4.** Longitudinal analyses \| \| --- \|  1. **Grey matter (GM)**  - **Interaction between times (BL – FU) and groups (controls > relapsers) : GM longitudinal comparison** | | | | | | | | | |
| --- | --- | --- | --- | --- | --- | --- | --- | --- | --- | --- |
| **Regions** |  |  | **Cluster level** | | **Peak level** | | **MNI coordinates** | | |
|  |  |  | k | p uncorr. | T | p uncorr. | **x** | **y** | **z** |
| L inferior frontal gyrus, triangular part |  |  | 183 | 0.036 | 5.34 | 2.22.10^-5^ | -39 | 46 | 12 |
| R inferior frontal gyrus, triangular part |  |  | 145 | 0.05 | 4.80 | 7.23.10^-5^ | 46 | 38 | 12 |

The height threshold was set at p < 0.001 uncorrected and the extent threshold at p <0.05 uncorrected; R = right, L = Left.

No significative difference for the:

- **Interaction between times (BL – FU) and groups (relapsers < controls) : GM longitudinal comparison**

| - **Interaction between times (BL – FU) and groups (controls < abstainers) : GM longitudinal comparison** |
| --- |

- **Interaction between times (BL – FU) and groups (controls > abstainers) : GM longitudinal comparison**

1. **White matter (WM)**

| - **Interaction between times (BL – FU) and groups (controls > relapsers) : WM longitudinal comparison** | | | | | | | | | |
| --- | --- | --- | --- | --- | --- | --- | --- | --- | --- |
| **Regions** |  |  | **Cluster level** | | **Peak level** | | **MNI coordinates** | | |
|  |  |  | k | p uncorr. | T | p uncorr. | **x** | **y** | **z** |
| R external capsule /Insula R |  |  | 261 | 0.035 | 5.64 | 1.19 10^-5^ | 33 | 8 | 10 |
| R external capsule R/inferior frontal gyrus |  |  |  |  | 4.31 | 2.12.10^-5^ | 27 | 24 | -3 |
| R anterior corona radiata |  |  |  |  | 4.05 | 3.78.10-4 | 24 | 26 | 0 |
| R anterior limb of internal capsule |  |  |  |  | 4.02 | 4.05.10^-4^ | 24 | 15 | 0 |
| L anterior limb of internal capsule |  |  | 173 | 0.078 | 4.45 | 1,54.10^-4^ | -21 | 16 | 3 |
| L external capsule /insula |  |  |  |  | 4.38 | 1.81.10^-4^ | -28 | 21 | 3 |

The height threshold was set at p < 0.001 uncorrected and the extent threshold at p <0.05 uncorrected; R = right, L = Left.

No significative difference for the :

| - **Interaction between times (BL – FU) and groups (controls < relapsers): WM longitudinal comparison** |
| --- |
| - **Interaction between times (BL – FU) and groups (controls < abstainers): WM longitudinal comparison** |

- **Interaction between times (BL – FU) and groups (controls > abstainers): WM longitudinal comparison**

**Figure 1.** Design of the longitudinal study : a 7-year follow-up

**Figure 2.** Whole brain analyses comparison between the present results and Kühn’s study.

A. Whole-brain analysis, Relapsers > Abstainers, at baseline and follow-up.

B. Whole-brain analysis showing a higher GM volume in bilateral caudate nuclei at age 14 as predictor of a stronger increase in alcohol use scores over 5 years, between age 14 and 19, with structural equation modeling (with permission from Kühn et al., figure from eLife 2019).


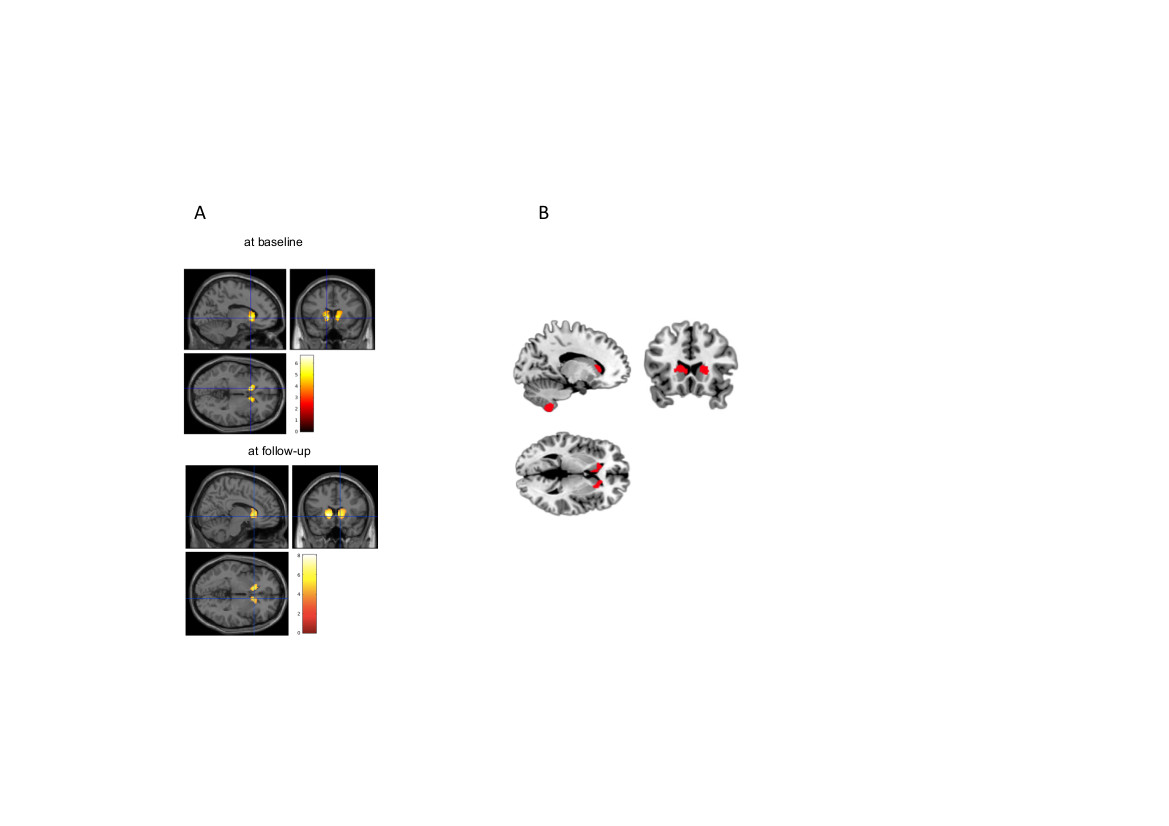

Supplement: Supplementary file 1 [file Data_Sheet_1.docx]
